# Advancements in Hypospadias Management: Trends, Techniques, Training, and Patient-Centric Outcomes

**DOI:** 10.5152/tud.2024.23219

**Published:** 2024-03-01

**Authors:** Vinaya Bhatia, Nicolas Fernandez, Christopher Long, Renea Sturm, Walid Farhat, Fardod O’Kelly

**Affiliations:** 1 Department of Urology, University of Wisconsin-Madison, School of Medicine and Public Health, Madison, WI, USA; 2 Department of Pediatric Urology, Seattle Children’s Hospital, Seattle, WA, USA; 3 Department of Pediatric Urology, Children’s Hospital of Philadelphia, Philadelphia, Pennsylvania, USA; 4 Division of Pediatric Urology, Mattel Children’s Hospital, University of California Los Angeles, Los Angeles, CA, USA; 5 Division of Pediatric Urology, Women and Children’s Hospital, University of Wisconsin School of Medicine and Public Health, Wisconsin, USA; 6 Division of Pediatric Urology, Beacon Hospital, University College Dublin, Dublin, Ireland

**Keywords:** Hypospadias, congenital disease, surgical simulation, complications, patient-reported outcomes

## Abstract

Hypospadias has drawn increasing attention due to its prevalence, complex etiology, and significant impacts on psychological and sexual quality of life. This comprehensive review delves into the facets of hypospadias management, exploring pivotal themes that shape present understanding and practice. We demonstrate potential explanatory factors for its incidence through an analysis of geographic, genetic, and environmental influences. We then contextualize care by exploring historical and evolving surgical techniques, and highlight that advances in surgical approaches employ a spectrum of repair strategies. Innovation in surgical training, with a focus on simulation-based methodologies, offers a bridge between didactic learning and real-world application, which is particularly relevant due to the demonstrated effect of personal experience with hypospadias repair outcomes. Considering the importance of mentorship, case exposure, and hands-on practice, a holistic understanding of expertise cultivation at each training stage is essential. Lastly, patient-centric outcomes research must take center stage, particularly as the impact of our interventions on children with hypospadias will be judged by them as adults. Shifting from solely surgeon-reported outcomes to patient-reported outcomes is emphasized in the review, allowing for a more comprehensive assessment of the influence of surgical interventions across the lifespan. Integrating patients’ perspectives refines surgical decision-making to align with expectations, ultimately enhancing overall satisfaction. Our multi-pronged exploration of advancements in hypospadias underscores the symbiotic relationship between evolving surgical techniques, training methodologies, personal experience, and patient-reported outcomes. As the field progresses, these insights will collectively contribute to optimizing hypospadias care, advancing both medical practice and patient well-being.

**Main Points**

- Possible contributors to the prevalence of hypospadias include geographic, genetic, and environmental influences.
- Innovations in surgical repair for hypospadias employ a wide spectrum of strategies, including grafted and staged approaches, with surgeon experience playing an important role in surgical success.
- Opportunities for improvement of surgical training include the use of biomaterials to create life-like models, virtual modeling, or active review of surgical videos by a surgical mentor with experience in hypospadias repair.
- Integrating patients’ perspectives into the surgical decision making process may allow surgeons to align results with patient expectations, and enhance overall satisfaction.

## Introduction

Hypospadias has drawn increasing attention due to its prevalence, complex etiology, and significant impacts on psychological and sexual quality of life. This comprehensive review delves into the facets of disease prevalence, etiology, and management and explores pivotal themes that shape present understanding and practice.

Through an analysis of geographic, genetic, and environmental influences, a clearer image emerges of the intricate interplay driving its incidence. Understanding evolving hypospadias repair techniques by tracing their historical trajectory to contemporary practices is invaluable in comprehending the present approach. Highlighting advances in surgical approaches, and opportunities to improve the learning curve through simulation, reveals a spectrum of strategies employed for hypospadias repair, while also assessing associated outcomes, complications, and patient satisfaction. Patient-reported outcomes may play an important role in allowing surgeons to closely and prospectively monitor patients over the long term. Herein, we present a comprehensive review of these considerations in the care of hypospadias.

## Prevalence, Trends, and Contributing Factors

There are two traditionally defined primary phases of phallic development, hormone dependent and hormone independent and each has potential genetic etiologies that can result in varied forms of hypospadias. To date, 49 genes have been reported to be associated with hypospadias development.^1^ Main categorizations of identified genes include those involved in genital tubercle and subsequent phallic development and urethragenesis, midline patterning, testis determination, androgen production and signaling, and susceptibility loci to environmental factors. Additionally, epigenetic mechanisms, long noncoding RNAs, miRNAs, unfolded protein response (autophagy-apoptosis), and metabolome likely are important in the interplay with genetic susceptibility and require future elucidation.^2^ Due to the range of associated genital development findings, phenotype/genotype correlation may be limited if meatal location is used as the main landmark to describe the phenotype. Proximal cases, for example, are more frequently associated with chordee, undescended testes, or genital ambiguity.

### Epidemiology

Hypospadias prevalence trends have been reported widely over the last decades.^3-6^ Multiple manuscripts have shown an increase in the prevalence over time, possibly related to environmental exposure to endocrine disruptors.^7^ Nonetheless, this increase has been questioned as possibly artifactual due to variations in methodological case definition, classification, and ascertainment.^8,9^ Yu *et al *published the results of a rigorous methodology that included analysis of data from 27 birth defect surveillance programs around the world. Prevalence was reported from 36 127 500 newborns. Worldwide prevalence was 20.9 per 10 000 newborns from 1980 to 2010. The reported increase was identified after 1999 with an increase of 0.25 new cases per year (*P* = .001).^4^ Available literature on regional differences has demonstrated geographical clustering with higher or lower prevalence in urban versus rural settings, and possibly an increased risk from high altitude levels.^4,10^

### Assisted Reproductive Techniques

The incidence and prevalence of hypospadias has been postulated to occur more frequently with the advent of assisted reproductive techniques (ART). An early study from Baltimore analyzing 41 patients over a 5-year period (1988-1992) indicated a 5-fold increased risk of hypospadias after in vitro fertilization (IVF), with an incidence of approximately 1.5% in the IVF group and 0.3% in the control group. The only recognized difference between the groups was maternal progesterone administration in the IVF group, but the exact cause of the increased risk of hypospadias was unknown.^11^ A subsequent Dutch case-control study assessed the impact of maternal diethylstilbesterol (DES) exposure in 8934 mothers and found a small but significant increased risk of hypospadias in the sons of women exposed to DES in utero compared with controls.^12^ A subsequent Hungarian study analyzing 890 neonates (1999-2008) conceived via IVF or intracytoplasmic sperm injection (ICSI) demonstrated the latter to be an independent risk factor for hypospadias in singletons (odds ratio, OR 3.19) and normal birthweight infants (>2500 g; OR 3.97).^13^ An epidemiological study from the Northern Netherlands analyzing the use of IVF/ICSI in sub-fertile couples reported an increased risk of penoscrotal hypospadias (amongst other anatomical abnormalities) associated with assisted reproduction with an OR of 13.49 but could not distinguish between IVF or ICSI as having a more putative effect.^14^ A large study from Massachusetts looked at 17 829 births arising from ART in both subfertile and fertile mothers. The group demonstrated a birth defect adjusted prevalence ratio of 1.5 for ART and 1.3 in subfertile compared with fertile deliveries. When performing a sub-analysis for hypospadias, the prevalence ratio was 1.8, and higher in singletons than in twins.^15^ A similar study which used the Centers for Disease Control (CDC) and Prevention’s Period-linked birth-infant death data files and fetal death database for 2011-2013 performed multivariate logistic regression to estimate the association between ART and birth defects. The group found amongst 71 050 pregnancies conceived by ART that the OR of developing hypospadias was 1.77 compared to controls.^16^

These findings were not isolated to mothers. A French study compared men prenatally exposed to DES with those who were not exposed and the general population. The authors described an increased transgenerational risk of hypospadias compared to controls with an OR of 22.9.^17^ A Japanese study using data from the Japanese-assisted reproductive technology registry (2007-2014) of fresh embryo transfer cycles (n = 59 971), described significantly higher incidences of hypospadias (OR 6.85) as a result of paternal subfertility compared to fertile men.^18^

### Familial-Isolated Hypospadias

There has long been described a genetic predisposition to the development of hypospadias with proposed increased incidences amongst siblings and offspring. Although there are multiple associations with other concurrent conditions and numerous genetic syndromes, there is a dearth of data which exists on isolated familial forms.

One of the earlier studies to describe this prospectively studied the families of 177 boys with varying degrees of hypospadias to determine the prevalence of hypospadias within this population. A significant number of male subjects in each family member category were affected, with first-degree relatives (brothers and fathers) having a 14% and 9% incidence, respectively. Siblings were at a greater risk when the proband had a more severe degree of hypospadias and with multiple family members affected.^19^ A study looking at genome-wide linkage in hypospadias assessed 360 microsatellite markers in 69 families with at least 2 members affected by hypospadias and found linkage regions in chromosome 9q22 in all families.^20^ A Dutch study of 405 cases of hypospadias and 627 controls (1987-1997) demonstrated that a family history of hypospadias increased the risk of offspring hypospadias, an effect that seemed to be more predominant in distal (OR 10.3) and midshaft (OR 9.2) sub-types.^21^

It is generally hypothesized that the family history of isolated hypospadias is under-reported and not captured effectively. A French multicenter study of 395 boys with hypospadias was prospectively screened for a family history with a questionnaire, clinical description, family tree, and sequencing of AR, SF1, SRD5A2, and MAMLD1 genes. A positive family history was identified in 22.3% of cases, with multiple events occurring in 19.3% of families. The results of genetic analysis combined with previous data on androgen receptor sequencing revealed that familial cases more frequently tended to demonstrate identifiable relevant genetic mutations as compared to sporadic cases.^22^ Click or tap here to enter text. Given these findings, consideration of including robust family history screening and possibly expanding genetic screening to distal and proximal hypospadias for familial cases may be advantageous to improve patient and family counseling.

## Evolution of Hypospadias Surgical Technique

Evaluation of the surgical management of hypospadias reveals a landscape that is under constant modification and evolution. There are over 300 described technical variations in the literature, most of which are variations upon an established technique. This evolution is driven by our desire to constantly improve outcomes for our patients. Given the differences in their management and severity of the disease process, we will divide these modifications by distal versus proximal hypospadias.

The Meatal Advancement and and GlanduloPlasty Incorporated procedure (MAGPI) represented a significant advancement toward surgeons demonstrating a higher expectation of cosmetic outcomes, specifically, a slit-like meatus within the glans, without the morbidity of a urethroplasty.^23^ Prior repairs, such as the island onlay flap, in which a dorsal preputial flap was rotated ventrally to facilitate urethral reconstruction, and the Mathieu repair, in which a perimeatal-based flap was used for urethral reconstruction, were often successful, but the cosmetic outcomes lacked consistency. However, long-term outcomes of the MAGPI when applied universally demonstrated higher complication rates, and it has since decreased in its indications and its use is much more limited than it once was.

The current technique of urethral tubularization with local tissue and subsequent glansplasty traces its origins to the Thiersch Duplay repair. This has since been modified with a midline incision, as described by Rich et al and eventually made a household name once coined the Snodgrass or Transurethral Incision of Plate (TIP) repair.^24,25^ Its original description indicated that any patient could be repaired by this approach by deeply incising the urethral plate a urethroplasty could be performed with any sized plate. Glansplasty would complete the slit-like meatus in hopes of creating a near perfect outcome. There have been significant modifications over time to improve the technique. First, the midline incision should be deep, down to the level of the corporal tissue. This will mobilize the urethra and will create a deep groove that can heal by secondary intention. The so-called “wide glans dissection” is now done to separate glans flaps, taking tension off of the tissue in order to allow closure without compromising the urethroplasty. The distal extent of the urethroplasty is now taken to what is often described as the “elbow” of the glans, which is thought to be the natural border of the urethral meatus. Doing so decreases the risk of meatal stenosis.^26^

The Thiersch Duplay repair differs from the TIP in that the local urethral plate is utilized without a midline incision and is not dependent on healing by secondary intention. As such, the width of the glans and the depth of the urethral plate play a key role in achieving a successful outcome. Preoperative testosterone administration in prepubertal boys increases glans width and has been shown to decrease complication development.^27^ Although its use has been controversial in the literature, there is an emerging data that suggests in select patients it improves outcomes.^28^

A modification of both the TIP and Thiersch Duplay is the dorsal inlay graft.^29,30^ This is an adaptation indicated for a poor urethral groove, which, when tubularized, poses a higher risk for complication development such as fistula formation, glans dehiscence, or wound breakdown. A deep midline incision, closed with an inlay graft of foreskin, has the benefit of creating a deep plate that will fold more easily and decreases the contracture risk when the plate is left to heal by secondary intention, particularly when the defect is large or the tissue is more dysplastic.^31^

Proximal hypospadias presents a unique challenge.^32^ Once originally designated only by the location of the urethral meatus, challenges with repair have expanded indications to include the degree of penile curvature, the quality of the penile skin, and the depth and width of the glans groove. Simply put, a proximal hypospadias can masquerade as a distal hypospadias and one must identify this to prevent significant patient morbidity.

A single-stage repair for proximal hypospadias was once thought to be the gold standard approach. It was justified in that the outcomes were thought to be better than that for a staged repair and achieved this goal in fewer surgeries. The single-stage TIP repair was expanded to include patients with more severe variants but follow-up indicated a higher complication rate for these patients, particularly the late development of recurrent curvature.^33,34^

Time has disproven this assumption and has led to a significant field change in management. One significant driver for this is penile curvature. What was once thought to be easily managed with dorsal plication alone has been met with high curvature recurrence rates. The importance of dividing the urethral plate and selective use of ventral lengthening procedures has emerged as a key element of curvature correction.^35^ Because of the significant long-term morbidity of persistent curvature, it is now a key element to correct at the time of the initial repair. The consequence of a more aggressive approach to diagnosis and correcting penile curvature, the multi-stage repair has emerged as the preferred approach toward optimal penile reconstruction.

The jury is still out on the optimal tissue selection for urethral reconstruction in staged repairs. Options include Byars flaps, island interposition flaps, and preputial or Buccal skin grafts. It is important to note that placing a skin graft over a ventral patch will lead to a higher skin graft contraction rate and is not recommended. Some have even advocated for a 3-stage repair when a ventral corporotomy is performed—the first stage the ventral corporotomies are covered with healthy dartos and skin, the second stage involves ventral preputial graft placement, while the third stage is the urethroplasty. Regardless of the approach, care of the graft or flap is geared toward even healing. Skin massage, with Vitamin E oil, silicon gel, or petroleum jelly, is thought to make a difference. Hyperbaric oxygen therapy and topical nitroglycerin have also been used to promote even wound healing as well.^36^

One must wonder why so many modifications exist, and what is clear is that there is no perfect hypospadias repair. Modifications are brought about because of one looking at their outcomes and adjusting their technique accordingly. There is no doubt that we still have room to grow, but we must also appreciate how far we have come.

This section delves into the critical role of surgeon experience and other factors in hypospadias repair outcomes, offering valuable insights into this complex surgical procedure. The evolution of hypospadias repair and minor technical modifications, including variables such as mobilization of the corpus spongiosum, urethral plate incision depth, or skin removal have a major impact on outcomes. Furthermore, it highlights the challenges associated with standardizing surgical approaches and the involvement of trainees in this intricate process.

### Factors Influencing Complications and Follow-up Care

Beyond surgeon experience, a broader context of factors influences complications and follow-up care in hypospadias repair. Hypospadias severity, surgical techniques, and penile curvature are among the key factors explored. This section underscores the importance of prolonged follow-up, especially for patients with proximal hypospadias and those at risk of late complications. Comprehensive care and early issue detection are essential components of successful outcomes.

## The Surgeon’s Journey: Phases of Mastery

The learning curve in hypospadias repair is a dynamic process marked by distinct phases, each with its unique characteristics and challenges. The CUSUM method is used in statistics to track and identify persistent shifts or changes in a process by calculating the cumulative sum of deviations from a reference value over time. This type of analysis has allowed us to identify these phases of surgical learning and their impact on surgical outcomes.^37^

### Learning Phase

In the initial phase, known as the learning phase, surgeons embark on their journey in hypospadias repair. This phase typically spans the first 127 cases. Surgeons encounter a range of complexities and nuances, learning the intricacies of the procedure. Operative time and complication rates during this phase exhibit considerable variation as surgeons adapt to different scenarios.

### Competence Phase

The competence phase represents a significant milestone in the learning curve. Beyond the 127th case, we observe a plateau in operative time and complication rates. Surgeons in this phase have honed their skills and gained confidence in their abilities. Surgical outcomes become more consistent, and the procedure becomes more routine.

### Proficiency Phase

The proficiency phase is the pinnacle of the learning curve, usually extending beyond the 234th case. Surgeons in this phase demonstrate a remarkable decline in operative time and complication rates. Their expertise allows for greater precision and efficiency in hypospadias repair. Notably, this phase is characterized by several key observations:

- Younger patients are often encountered, indicating a growing comfort level with challenging cases from a young age.
- More severe degrees of distal hypospadias are confidently repaired using the tubularized incised plate approach, showcasing the surgeon’s mastery of various techniques.
- Complications related to meatal stenosis and cosmetic issues become increasingly rare, underscoring the surgeon’s ability to deliver consistently successful outcomes.

The CUSUM methodology provides valuable insights into the learning curve in hypospadias repair, highlighting the progression through learning, competence, and proficiency phases. This journey underscores the crucial role of surgeon experience in reducing complications and achieving optimal results. As surgeons advance along this curve, they not only refine their technical skills but also develop the confidence to tackle increasingly complex cases, ultimately benefiting patients with hypospadias. This understanding of the learning process is a fundamental aspect of improving outcomes in hypospadias repair.

Understanding the learning curve in hypospadias repair may differ among surgeons, especially in proximal cases. A comprehensive analysis of 73 primary hypospadias repair cases conducted by a single pediatric urologist reveals the significant impact of surgeon experience on outcomes.^38^ This retrospective study demonstrates that the learning curve stabilizes after approximately 50 cases, marking a crucial turning point in achieving successful proximal hypospadias repair. However, supervision by experienced surgeons remains vital during the early phases of the learning curve.

### Ensuring Comprehensive Care and Ongoing Monitoring

Short follow-up periods can lead to the missed detection of late-onset complications, and this review echoes the importance of advocacy for ongoing monitoring and care for all patients who undergo hypospadias repair.

In summary, this overview demonstrates the importance of surgeon experience and synthesizes findings from multiple studies, offering insights for both medical professionals and patients. Our findings underscore the importance of continuous research, comprehensive follow-up care, and tailored training approaches to ensure the best possible outcomes for patients with hypospadias. One area of emerging research that could inform this work and help decrease the learning curve is related to surgical simulation.

## Exploring the Role of Simulation-Based Training

Current mentor–mentee intraoperative training models allow for limited exposure to varied approaches and instrumentation.^39^ Effective training opportunities are needed to disseminate the specific details of surgical techniques, decrease the surgical learning curve,^37^ and aid surgeons in determining the applicability of each approach to individual patient phenotypes.

Along the timeline of surgeon development from student to experienced clinician, there are several key points in hypospadiologist training that could benefit from augmented or hybrid simulation in addition to intraoperative training options.^40^ The learning phase of training,^37^ as described in the earlier section, encompasses residency, fellowship, and early practice. In medical school and early residency, this phase focuses on procedural understanding, suturing, and tissue handling techniques. In later residency and fellowship, procedural planning (including decision-making) regarding varied phenotypes and penile tissue coverage becomes increasingly critical. As fellowship progresses into early surgical practice, a major goal is to improve early outcomes. Finally, as novel techniques and devices emerge throughout a surgical career, creating learning models to facilitate ongoing process improvement could decrease the time for advancement and skill acquisition in this field. Each of these stages may require differing levels of model fidelity and repetition to meet learner needs.

### Considering Trainee Level

Selection of training material requires consideration of trainee level, background, and pre-existing skill set (Table 1). For example, a recent review evaluated the optimal approach to acquisition and retention of suturing skills for medical students.^41^ Dry (low-fidelity) models were as effective as wet (tissue based) models for both learning and retention of suturing skills.^42^ There was an additional benefit to video-based skills teaching to complement this, particularly if this was viewed as a refresher throughout the learning period. Skill acquisition was not affected by the level of trainer; trained senior medical students provided equivalent results when providing supervision when compared to senior surgeon mentors.^43^ As one establishes a hypospadias training curriculum, it will be important to consider a framework that enables both early skill acquisition and sustained training opportunities. A program that provides a lower fidelity practice model that can be used repeatedly may more effective for skills retention than higher fidelity surgical models.^44^

As residents and fellows progress, their needs change asynchronously due to varied surgical experience and individualized learning curves for each skill (Table 1). Despite many pediatric urologists reporting a lack of resident participation beyond an assistant level in hypospadias repair, step-wise defined participation of senior residents during a tubularized incised plate urethroplasty has demonstrated equivalent overall outcomes to those described in published literature.^45^ This approach to step-wise training also describes the importance of communication regarding case goals, with a focus on individualized training levels. A concern remains that surgeon-mentee individualized skills assessment varies widely and may fail to address focused areas for improvement. In other fields, machine learning methods have been used to predict need areas and learning curves for each individual.^46^ When specific areas for improvement are recognized, there are multiple developing modalities that may increase repetition and exposure. A combination of low- and high-fidelity models at this level that are focused on the acquisition of specific techniques may decrease this learning curve. In one series, in which junior and senior residents were randomized to undergo low-fidelity tasks versus intermediate-fidelity hypospadias repair on a silicone model, outcomes of the repair were improved in those who had completed the low-fidelity tasks first, prior to undertaking the silicone model hypospadias repair. This finding did not change by trainee level (junior or senior).^44^

### Considering Tissue Selection

When desired, selection of materials or tissue with tensile-matched properties and multilayered design could increase fidelity and improve surgical skill acquisition at the resident trainee level, as has been observed in microvascular anastomoses by re-creating natural tissue planes and mechanical properties of healthy tissue. Examples of this include the use of crosslinkable hydrogels such as PVA which are flexible and suturable and can be used in scaffolds to mimic skin.^47^ It is anticipated that the goals of skill acquisition can be met through a combination of modalities, enhancing training by providing a needs-based program that facilitates procedural understanding and skills repetition.

### Considering Early Career Surgeons

As one progresses through training, personalized skills refinement becomes increasingly invaluable. Methods currently used in a robotic or laparoscopic environment are simulation-based training, formal curriculum, and mentorship. Put together into a robotic curriculum, these methods have substantially decreased learning curves in complex robotic procedures.^48^ Adding a level of formal review to hybrid training models in trainee-mentee dyads may further enhance performance across individuals. For skills acquisition and maintenance, higher fidelity models may also be invaluable to determine depth of urethral plate or corporal incisions, while longer term maintenance of urethroplasty techniques may only require a lower fidelity model. Additionally, a technical aspect that may be unique to this level is the value of 3-dimensional visualization (e.g., VR, 3D video clips) which has been demonstrated in the laparoscopic environment to aid in both 2D and 3D skills acquisition when compared to training in a 2D environment for novice surgeons (e.g., video/photo), an effect that was not seen in experienced surgeon participants.^49^ Likewise, robotics has demonstrated that haptic feedback may play differing roles with experience, another key area that is difficult to train in a traditional manner as one becomes experienced with the finer aspects of wound tension.^50^ Finally, although it may not be immediately feasible with current development costs and technology, future platforms that provide enhanced feedback regarding technical decisions and tissue reconstruction, such as those portrayed in a 3D video or VR/AR model, could facilitate repeated trials to ensure exceptional cosmetic results regardless of patient phenotype.^51^

### Ongoing Skills Development

Surgical skills acquisition does not terminate at the end of formal training.^37^ While it is not known what this maintenance curve would be for proximal hypospadias, a recent network study indicated that >60% of pediatric urologists performed 10 or fewer proximal hypospadias repairs per year. To increase their exposure to these cases, many pediatric urologists review online videos such as those available on YouTube distributed through the Hypospadias Specialty Center (PARC Urology). Broader development of online video sharing could further enhance the understanding of the range of techniques and approaches currently in practice for hypospadias repair. Additionally, surgical mentorship for complex cases has been suggested, with dual cases performed by senior and junior surgeons in tandem by using either telementoring or in-person strategies. The combination of resultant video libraries may ultimately benefit from being linked to patient phenotype through video processing and machine learning, allowing one to provide detailed anatomic descriptors or a 3D model of their patient’s phenotype to guide their use of a shared video resource to visualize individualized surgical repair options. Despite ongoing rapid advancements in technology and options to support surgical training and skills maintenance, significant challenges remain, including the production of high-quality videos without significant additional cost, support of mentor-mentee time for process and skills improvement, and ensuring that methods are validated across surgical skill levels and needs. Ultimately, to be successful, we envision that hypospadias repair must be viewed as an area of community engagement, with necessity for multi-institutional mentorship and technical improvement across surgeon needs and patient phenotypes.

## Considering Repair from the Patient Perspective: The Role for Patient-Reported Outcomes in Long-Term Surveillance and Surgical Decision-Making

Patient-Reported Outcomes (PROs) in hypospadias have been extensively explored and vary in their respective focus areas,^52^ including comparison of surgeon and patient priorities in cosmesis, studying the varying effects of disease complexity on psychosocial health, assessment of urinary symptoms,^53^ and to help patients and providers determine the effects of differing complications, functional outcomes, and surgical technique selection.^54-56^ One particularly innovative approach to incorporating the patient perspective into care paradigms is centered on a formal Decision Aid. This aid encompasses materials written in lay language about hypospadias, testimonials from families who have undergone repairs, and a functioning “help me decide” tool, which has demonstrated high user acceptability and usability in pilot testing.^56^

Further, current studies on long-term outcomes paint a worrisome picture of missed opportunities to provide holistic care for patients from a surgical and psychosexual perspective. Adult data demonstrates that up to 40% of men will report lower urinary tract symptoms,^57^ while at least half of patients may have sexual problems including erectile or ejaculatory dysfunction.^52^ A recent study focusing specifically on adolescent outcomes demonstrated that psychosexual health was closely correlated with patient’s satisfaction with their overall penile appearance.^58^ Late presenting functional complications are a known entity that have been well-described in several long-term series after distal and proximal repair.^33,58-60^

Therefore, surgeons need better tools to holistically assess concerns as patients mature. To facilitate the development of improved PROs, a conceptual model for hypospadias-specific HRQoL was proposed based upon thematic coding of existing literature and includes domains of penile appearance, voiding function, social function, psychological health, and sexual health.^52^ When looking at existing PROs in hypospadias, many measures have been used to explore penile appearance, voiding function, and sexual health, but no single measure comprehensively explores all proposed domains.^61^ Ongoing studies are revising this conceptual model based upon patient and parental input, and future studies will focus on the development of a dedicated HRQoL measure.^53^ Existing measures in hypospadias have made important strides in determining patient priorities related to hypospadias surgery. Available measures that have been developed and validated for hypospadias include the following.

### Penile Appearance-Focused Measures

The Genital Perception Scale (GPS)^61^ was the first PRO to be developed in the hypospadias population. The measure reviewed glans anatomy, penile girth, flaccid penile length, and appearance of the scrotum and testes. It was validated in two forms, a pediatric form and an adult form, and demonstrated that proximal hypospadias patients tended to have a lower GPS score. The Pediatric Penile Perception Scale/Penile Perception Scale (PPPS/PPS)^61^ was dedicated to obtaining the patient’s opinion on post-operative appearance. This measure focuses solely on aspects of penile appearance, including general appearance, meatal position, glans anatomy, and shaft skin. It has been used in several other studies as a measure of technical outcomes after hypospadias repair.^61^

### Penile Appearance and Voiding Measures

The Hypospadias Objective Score System (HOSE)^61^ was a 5-point measure designed to compare parent-proxy PROs focused on penile appearance to estimations of surgeons and RNs. In addition to an assessment of penile appearance, the authors included a question on voiding function to increase utility. Its minimal response burden and wide applicability have led to its use in multiple studies of surgical technique. The mini-International Prostate Symptom Score (mini-IPSS)^61^ was a measure designed to assess penile appearance, voiding function, and parental worry. The development of this measure was based upon priority domains identified after an extensive literature review, open-ended interviews from families and patients, and focus groups. However, this measure was only determined to have face validity, and did not complete further reliability and validity testing. The measure was translated into English and Spanish and has been used in multiple studies reviewing postoperative preferences.

### Psychological Health Measures

To date, no generic psychological measure has been valid in the hypospadias population.^61^ It is possible that disease-specific measures may be required to meaningfully measure the psychological impact of hypospadias and hypospadias treatment.

### Sexual Health Measures

The Satisfaction in Genital Hypospadias Treatment (SIGHT) is a German questionnaire designed to assess psychosexual development in adolescents who had previously undergone hypospadias surgery. Discriminant validity was demonstrated for surgical complications, but this measure has not been adapted to an English-speaking population or used in additional studies of psychosexual outcomes after surgery.^61^

*Hypospadias-Specific Measures undergoing validation:* One measure for hypospadias that is fully based on qualitative interview data from patients, caregivers, lay, and medical experts is undergoing continued validation and will allow for re-evaluation of patient outcomes from the patient and caregiver-proxy perspective.^53^

Ongoing work is promising for the development of HRQoL measures and tools to enact recommendations based on the findings of these measures that will adaptively measure and respond to the needs of patients as they reach sexual maturity. A patient-prioritized approach to long-term follow-up, rather than a purely algorithmic approach, may be needed to adequately address the complex functional, cosmetic, and psychosocial and psychosexual concerns that arise after hypospadias repair.

## Conclusion

The care of hypospadias has evolved significantly over the past several decades, but more work remains to be done. Next frontiers in care will involve identifying and addressing risk factors in the prenatal setting, identifying and creating repositories of the most successful surgical techniques for dissemination, creating opportunities for technical improvement through simulation and repetition for learners at all levels, and fostering improved approaches to patient-prioritized follow-up. We hope that this review will serve as a meaningful reference to surgeons dedicating themselves to these important efforts.

## Figures and Tables

**Table 1.** Proposed Model Fidelity and Materials by Trainee Level

Trainee Level	Recommended Fidelity Level	Citation	Materials Used
Medical students	Low	Denadai 2012	Videos
Junior residents	Low Low, followed by Intermediate	O’Connor 2018 Bhatia 2022 Atlan 2018	Computer module Silicone Polyvinyl alcohol gelatin
Senior residents	Low Low, followed by Intermediate	O’Connor 2018 Atlan 2018 Bhatia 2022	Computer module Silicone Polyvinyl alcohol gelatin
Early career surgeons	High, with mentorship	Rice 2020 Sorenson 2017	Virtual reality Animal tissue Tensile-matched/layered synthetic constructs
